# Predictors of fetal anemia and cord blood malaria parasitemia among newborns of HIV-positive mothers

**DOI:** 10.1186/1756-0500-6-350

**Published:** 2013-09-03

**Authors:** Amos K Laar, Fredrick E Grant, Yaw Addo, Ireneous Soyiri, Bright Nkansah, James Abugri, Alexander S Laar, William K Ampofo, Juliette M Tuakli, Isabella A Quakyi

**Affiliations:** 1 Department of Population, Family, & Reproductive Health, School of Public Health, College of Health Sciences, University of Ghana, Box LG 13, Legon, Accra, Ghana; 2 International Potato Centre, Mama SASHA Project, P.O. Box 1879, Bungoma, Kenya; 3 Hubert Department of Global Health, Rollins School of Public Health, Emory University, Atlanta, GA, USA; 4 School of Public Health, University of Ghana, Accra, Ghana; 5 Global Public Health, School of Medicine & Health Sciences, Monash University, Sunway Campus, Malaysia; 6 Nii Boi Town, Box NB 1157, Accra, Ghana; 7 Department of Applied Chemistry and Biochemistry, Faculty of Applied Sciences, University for Development Studies, Navrongo Campus, Ghana; 8 Project Fives Alive! National Catholic Health Service, Health Directorate, Lamashegu, Tamale, Ghana; 9 Noguchi Memorial Institute for Medical Research (NMIMR), University of Ghana, Legon, Ghana; 10 Department of Biological, Environmental, & Occupational Health Sciences, School of Public Health, College of Health Sciences, University of Ghana, Box LG 13, Legon, Accra, Ghana

**Keywords:** HIV, Malaria, Fetal anemia, Cord blood malaria parasitemia, Ghana

## Abstract

**Background:**

Malaria and HIV infections during pregnancy can individually or jointly unleash or confound pregnancy outcomes. Two of the probable outcomes are fetal anemia and cord blood malaria parasitemia. We determined clinical and demographic factors associated with fetal anemia and cord blood malaria parasitemia in newborns of HIV-positive women from two districts in Ghana.

**Results:**

We enrolled 1,154 antenatal attendees (443 HIV-positive and 711 HIV-negative) of which 66% were prospectively followed up at delivery. Maternal malaria parasitemia, and anemia rates among HIV+ participants at enrolment were 20.3% and 78.7% respectively, and 12.8% and 51.6% among HIV- participants. Multivariate linear and logistic regression models were used to study associations. Prevalence of fetal anemia (cord hemoglobin level < 12.5 g/dL) and cord parasitemia (presence of *P*. *falciparum* in cord blood at delivery) were 57.3% and 24.4% respectively. Factors found to be associated with fetal anemia were maternal malaria parasitemia and maternal anemia. Infant cord hemoglobin status at delivery was positively and significantly associated with maternal hemoglobin and gestational age whilst female gender of infant was negatively associated with cord hemoglobin status. Maternal malaria parasitemia status at recruitment and female gender of infant were positively associated with infant cord malaria parasitemia status.

**Conclusions:**

Our data show that newborns of women infected with HIV and/or malaria are at increased risk of anemia and also cord blood malaria parasitemia. Prevention of malaria infection during pregnancy may reduce the incidence of both adverse perinatal outcomes.

## Background

Malaria in pregnancy may be second to only HIV in terms of mediation of adverse maternal and perinatal outcomes [1,2]. These infections during pregnancy can individually or jointly unleash or confound these outcomes. The enormity of the burden of fetal anemia, defined as cord hemoglobin level < 12.5.0 g/dl, is huge with reportedly very high prevalence in sub-Saharan Africa. Prevalence of fetal anemia ranging from 23 – 66% have been observed in studies in Malawi [3] and Nigeria [4] with one study in Mozambique documenting up to 93% of newborns [5].

There is a general consensus that HIV-uninfected or immunocompetent HIV-infected women with relatively good levels of CD4+ T cells (> 350 cells/μl of blood) can mount protective immune responses when exposed to malaria infection, and this can limit malaria and other infections [6,7].

Malaria infection (at low density) has been shown to induce and sustain the production of the protective cytokine Interferon gamma (IFN-γ), facilitate protective immune factors such as Leukocyte Inhibitory Factor (LIF), all of which, can reduce HIV-1 replication [8]. Women with low levels of CD4+ T cells (< 350 cells/μl) however, have a reduced ability to mount protective immune responses to either malaria or other infections. HIV-infection severely impairs both interleukin-12 (IL-12) and cytokine interferon gamma (IFN-γ) pathways, leading to greater susceptibility to malaria infection in pregnant women [9]. Women with suppressed immune system due to HIV- infection are more likely to develop severe clinical malaria and other opportunistic illnesses, which can favor overproduction of cytokines such as Tumor Necrosis Factor alpha (TNF-α) [9,10]. This will, in turn, enhance HIV-1 replication, culminating in adverse outcomes both in the woman and her unborn baby [10] some of which could result from compromised materno-fetal substance transfer system.

It has been hypothesized that cord blood may become infected with malarial parasites through maternal transfusion into fetal circulation either at the time of delivery, during pregnancy, direct penetration through the chorionic villi, and or premature separation of the placenta [11]. With respect to malaria parasites, other investigators have argued that the placenta is remarkably capable of restraining the passage of the Plasmodium species to the fetus [12], while others [2,9] have suggested an ability of the fetus to resist infection. This resistance, may reflect among other things the physical barrier of the placenta to infected red cells, the passive transfer of maternal antibodies, and the poor environment afforded by the fetal red cells for plasmodial replication [2,9-12].

Studies from some African countries have shown the relationship between cord parasitemia and other adverse perinatal events but this has been hindered by data paucity. Villamor *et al*. [13] for example examined the risk of adverse perinatal outcomes in relation to maternal or umbilical cord *Plasmodium falciparum* parasitemia among HIV-infected women from Tanzania and found factors associated with cord parasitemia included maternal parasitemia at the first antenatal visit, and at delivery but not CD4 cell counts, parity, and zinc supplementation. Related reports from both malaria-endemic and non-endemic areas show higher prevalence of cord malaria ranging from 8% to 64.6% [14-17]. The condition in some cases has been shown to be strongly associated with placental malaria [18], thus increasing drug resistance, virulence of the parasite, or HIV [19].

Reduced cord blood hemoglobin concentration or fetal anemia, malaria is thought to play a role through a combination of systemic and local effects [20]. Systemic effects may be mediated through malaria-induced maternal anemia and local effects through placental malaria infection [21-23]. While there is currently no agreement as to the main mechanisms mediating malaria-associated fetal anemia [20], severe or chronic infection and the associated cellular immune responses may result in the consumption of glucose and oxygen that would have gone to the fetus. Histopathologic studies of malaria-infected placentae have also found thickening of the cytotrophoblastic membranes which may interfere with nutrient transport to the fetus, subsequently leading to fetal anemia [20,24].

The contributory role of maternal malaria infection in fetal anemia has been evaluated in a number of studies with varying results. A Malawian study found a higher prevalence of fetal anemia to be associated with increasing peripheral and placental parasite densities [3]. Other studies as reviewed by McElroy et al. [25] have found no statistically significant connection between evidence of malaria infection and fetal anemia [25]. A study of pregnant Turkish women found no significant difference in mean cord hemoglobin levels of neonates of anemic mothers compared to those of non-anemic mothers [26]. Relatively higher prevalence of fetal anemia were observed among babies born to malaria infected women, HIV-infected women and anemic women even though the differences were not statistically significant [27].

Based on these discrepancies in evidence, the predictors of cord malaria parasitemia, and infant anemia among infants of HIV-positive with malaria are worthy of investigating. We explore maternal anemia at first antenatal visit, maternal anemia at delivery, maternal malaria infection at first antenatal visit, and at delivery, maternal malaria infections both at first antenatal visit and at delivery, maternal CD4+ T cell count, teen pregnancy as potential predictors of fetal anemia and umbilical cord parasitemia for malaria.

## Methods

### The study sites

This study was conducted at three hospitals in Ghana – the Tema General Hospital in the Tema Municipality, Greater Accra region, Atua Government Hospital and St Martin *de Porres* Hospital both in the Manya Krobo District, Eastern region.

### Study design, population, participants and summary of field procedures

This prospective study design involved pregnant women seeking antenatal care services at three public hospitals. The women who met the study’s inclusion criteria and consented to enroll in the study were recruited. When these women returned to deliver at their designated hospitals, follow up data on perinatal outcomes including fetal hemoglobin concentration and cord blood malaria parasitemia were measured. Overall 1,154 (443 HIV-positive and 711 HIV-negative antenatal attendees), 171 with malaria, and 983 without malaria were recruited. At their first antenatal visit, we collected data on the background, socio-demographic characteristics, obstetric, and reproductive history of the study participants. Complete physical and clinical appraisals were carried out by the experienced nurses/midwives, and 5 ml blood samples collected by phlebotomists. Seven hundred and sixty-one (295 HIV-positive and 466 HIV-negative) of the 1154 women had their follow up data at delivery taken. Details on the study’s inclusion and exclusion criteria, sample size determination, sampling and follow up procedures have been published elsewhere (Laar et al., 2010).

### Ethical issues

The research protocol met the guidelines for research involving human subjects of the Noguchi Memorial Institute for Medical Research (NMIMR). The study protocol was first reviewed by the Proposal Review Committee of the School of Public Health, University of Ghana for appropriateness and scientific content. An ethical clearance was afterwards obtained from the Institutional Review Board (IRB) of the Noguchi Memorial Institute for Medical Research, University of Ghana, Legon. During field data collection, all eligible prospective women were counseled by trained nurse counselors, who were informed about the purpose of the study. Written informed consent, for those who were literate and witnessed verbal informed consent for the illiterate were obtained from the study participants. Subjects were informed about the objectives and methods of the study. They were also assured of strict confidentiality with regards to any information obtained from them.

### Laboratory measurements

Blood samples (5 ml) were collected from participating women at two time points. Venous blood was taken both at recruitment and at delivery; cord blood was taken only at delivery into heparinized EDTA vacutainers for analysis.

### Maternal HIV status at recruitment

HIV infection was determined using the Determine® HIV-1/2 Rapid Test Kit (Abbott Laboratories Diagnostics Division, IL, USA). It is an immunochromatographic test for the qualitative detection of antibodies to HIV-1 and HIV-2. Blood sample is added to the sample pad. As the blood migrates through the conjugate pad, it reconstitutes and mixes with the selenium colloid-antigen conjugate. This mixture continues to migrate through the solid phase to the immobilized recombinant antigens and synthetic peptides at the patient window site. If antibodies to HIV-1 and or HIV-2 are present in the blood sample, the antibodies bind to the antigen-selenium colloid and to the antigen at the patient window, forming a red line at the patient window site. If antibodies to HIV-1 and/or HIV-2 are absent, the antigen-selenium colloid flow past the patient window and no red line is formed at the patient window site.

### Maternal and cord malaria parasitemia

Malaria parasitemia was determined using Rapid Test Kit (Paracheck Pf, Orchid Biomedical Systems, India) that detects the presence of *P*. *falciparum*-specific protein (Pf. HRP-2) in whole blood specimen up to 14 days after the infection has been cleared. This test also utilizes the principle of immunochromatography. As the test sample flows through the membrane assembly of the dipstick after placing into the clearing buffer tube, the colored anti Pf HRP-2 antisera-colloidal gold conjugate (monoclonal) complexes the Pf HRP-2 in the lysed sample. This complex moves further on the membrane to the test region where it is immobilized by the anti Pf HRP-2 (monoclonal) antisera coated on the membrane leading to formation of a pink colored band which confirms a positive test result. Absence of this colored band in the test region indicates a negative test result, with a control band that serves to validate the test performance.

### Maternal and cord blood hemoglobin concentration and maternal CD4+ count

Maternal venous blood and infant cord blood hemoglobin concentrations were determined using an Automated Hematologic Analyzer (which measures hemoglobin by the formation of hemoglobincyanide). In addition the analyzer also directly measures the cell count for total red blood cells, white blood cells, and platelets. Maternal CD4+ count was determined using the Becton Dickinson (BD) FACScount system, which measures absolute CD4 counts (Immunocytometry Systems, San Jose, CA).

### Birth weight and gestational length

A research midwife weighed infants to the nearest 10 g on a standard seca scale immediately after birth. Gestational length was determined by last menstrual period of the study participant.

### Data analysis

Statistical analyses were done using SPSS Version 15.0 (SPSS Inc. Chicago, Illinois). Preliminary assessments of normality of the distributions of relevant continuous outcome variables were done using normal probability plots. Appropriate measures of centrality and of dispersion as well as frequencies were then computed. Proportions of various outcomes were compared using the the χ2 test. For comparisons of means of relevant continuous variables by maternal HIV-infection status, the Independent T-test was used.

The associations between cord blood malaria parasitemia status, cord blood hemoglobin concentration and maternal demographics and clinical outcomes were investigated using multivariate regression models. The strength of associations for binary outcomes was estimated using logistic regressions and presented as odds ratios (ORs) and their 95% confidence intervals (CI). The confounders and other covariates used in the multiple regression models were included based on a theoretical framework and previous studies (refs). The two main outcomes were treated as follows:

As binary outcomes, cord malaria parasitemia (0 – malaria parasitemia absent; 1 – malaria parasitemia present) fetal anemia (0 – cord blood hemoglobin less than 12.5 g/dL; 1 – cord blood hemoglobin concentration is greater than 12.5 g/dL). Covariates for these dichotomous outcomes included maternal anemia at recruitment (per the three definitions given earlier): anemia, moderately severe anemia, and severe anemia); maternal anemia at delivery with the same categories, maternal malaria at recruitment (0, 1), and maternal malaria at delivery (0, 1). As a continuous outcome variable, cord blood hemoglobin concentration were related with birth weight, gestation length, sex of new born, and maternal parasitemia status in cord or mother. Two-sided p-values < 0.05 were considered statistically significant.

## Results

The characteristics of study participants (HIV-positive and HIV-negative women) and their infants are shown in Table 1 and Table 2. The mean (SD) age of the mothers was 28 years (Table 1) with a majority of them aged 25 years or older (Table 2). HIV- women were significantly younger but heavier than their HIV+ counterparts, but did not differ significantly by body mass index (BMI) – see Table 1. Related pre-delivery characteristics by maternal HIV-status are presented in Table 2. Such bivariate analyses revealed significant differences in both maternal and infant characteristics/outcomes (Table 2).

**Table 1:** **Pre**-**delivery characteristics of HIV**-**infected and**, **HIV**-**uninfected women**, **as well as infant outcomes at birth** (**n at birth** = **295 mother**-**infant pairs**)

	**N**	**Mean**	**SD**	Mean difference*	**95% CI of the difference**	
**Mother**						
Age (years)	440	28.5	5.6	−2.1	−2.8	−1.4
Weight (kg)	440	62.5	11.4	3.0	1.4	4.6
Height (cm)	440	159.6	12.2	−1.3	−2.8	0.3
BMI (kg/m2)	440	25.8	16.4	2.9	−0.8	6.6
Hemoglobin level at recruitment (g/dL)	440	9.8	1.7	1.3	1.1	1.4
**Infant**						
Gestational age	295	37.6	2.1	0.7	0.4	1.0
Birth weight	281	2.8	0.5	0.2	0.1	0.3
Fetal Hemoglobin	295	11.7	2.7	−0.9	−1.3	−0.5

*The “means” and “standard deviations” presented in the table are those of HIV+ women; Mean difference = Mean of HIV-negative minus Mean of HIV-positive.

**Table 2:** **Pre**-**delivery characteristics of study participants and infant outcomes at birth by maternal HIV**-**status**

**Mother**	**HIV+**	**HIV-**	**Chi square**	**p value**
Maternal anemia	232 (78.7)	350 (51.6)	0.368	0.832
Maternal malaria	60 (20.3)	91 (12.8)	5.645	0.018
Married or cohabiting	363 (82.5)	476 (66.7)	34.390	<0.001
Up to 9 years of formal education	236 (53.6)	525 (73.8)	49.521	<0.001
Rural residence	333 (75.7)	431 (60.5)	27.937	<0.001
19 years or younger	20 (4.5)	94 (13.2)	37.734	<0.001
20-24 years	85 (19.3)	191 (26.8)		
25 years or older	335 (76.1)	427 (60.0)		
First trimester	35 (8.0)	73 (10.3)	5.938	0.051
Second trimester	184 (41.8)	329 (46.5)		
Third trimester	221 (50.2)	305 (43.1)		
Primigravid	129 (29.3)	228 (32.0)	5.509	0.064
Secundigravid	157 (35.7)	207 (29.1)		
Multigravid	154 (35.0)	277 (38.9)		
Nulliparous	9 (2.9)	273 (38.9)	156.332	<0.001
Primiparous	173 (55.6)	183 (26.1)		
Secundiparous	73 (23.5)	138 (19.7)		
Multiparous	56 (18.0)	107 (15.3)		
CD4-count at recruitment < 350 cells/mm3	133 (45.1)	-	-	-
CD4-count at recruitment < 200 cells/mm3	78 (26.4)	-	-	-
**Infant**				
Anemia (Hb < 12.5 g/dL)	169 (57.3)	319 (71.7)	16.374	<0.001
Female sex,	121 (41.0))	256 (54.4)	12.924	0.002
Preterm delivery	72 (24.4)	67 (14.5)	11.77	0.003
Low birth weight 1	63 (21.4)	67 (14.7)	7.071	0.029
Cord malaria present	72 (24.4)	52 (11.7)	20.950	<0.001

About a quarter (24.4%) of all babies born to HIV+ women were delivered before term (< 37 weeks gestation) with mean (SD) gestational age of 37.6 (2.1) weeks and majority (59%) of the infants were boys. The mean (SD) hemoglobin level of these infants at birth was 11.7 (2.7) g/dl and the prevalence of fetal anemia was also high (40%). The prevalence of cord malaria parasitemia among these group of HIV-exposed babies was 24.4% and 22.4% of them were low birth weight. Related statistics for their HIV-unexposed counterparts are detailed in the same table (Table 1 and Table 2).

Using multiple regression analysis, the factors associated with fetal anemia, and also with cord malaria parasitemia status are examined (Table 3). Fetal cord hemoglobin status at delivery was positively and significantly associated with maternal hemoglobin and gestational age (marginal significance) whilst female gender of infant was negatively associated with cord hemoglobin status, such that female newborns had, on the average 1.05 g/dl Hb lesser than their male peers. Maternal malaria parasitemia status at recruitment and female gender of infant were positively associated with fetal cord malaria parasitemia status. A separate sub-analysis HIV+ mothers and their HIV-exposed newborns revealed that CD4 count was not significantly associated with fetal cord hemoglobin or with fetal malaria (p > 0.05 in each case) (data not shown).

**Table 3:** **Maternal pre**-**delivery and infant predictors of cord hemoglobin concentration and malaria parasitemia of infants of HIV**-**positive mothers**

**Variable**	***Coefficients*, *β *(*95*% *CI*) *for predicting fetal cord Hb*, *g*/*dl***	**p**-**value**
*Fetal cord Hb*, *g*/*dl*, (*n*=*295*)	0.29 (0.03, 0.55)	0.029
Maternal Hb (g/dL)	−1.05 (−1.66, -0.44)	0.001
Infant sex (male=0, female=1)	0.21 (−0.01, 0.43)	0.066
Gestational age (wks)	−0.29 (−1.12, 0.54)	0.048
Maternal malaria status		
	***Odds ratio *(*95*% *CI*)**	
	***for predicting fetal cord malaria parasitemia***	
*Fetal cord malaria parasitemia*, (*n*=*295*)		
Maternal malaria (no=0, yes=1)		
Infant sex (male=0, female=1)	0.42 (0.21, 0.84)	0.014
Maternal anemia	1.97 (1.06, 3.66)	0.032
Nine or more years of education	1.07 (0.909, 1.26)	0.067
	1.20 (0.68, 2.10)	0.089

Follow up presented in Table 4 respectively identified gestational age and maternal malaria status as predictors of fetal anemia and cord blood malaria parasitemia. Odds of fetal malaria parasitemia for for both HIV status = negative and HIV status=positive. The multivariable logistic regression revealed “malaria status” was statistically significant in both instances.

**Table 4:** **Maternal pre**-**delivery and infant predictors of cord hemoglobin concentration and malaria parasitemia of infants of HIV**-**negative mothers**

		***95% CI for predicting fetal cord Hb, g/dl***		
	***Coefficients, β***	**Lower bound**	**Upper bound**	**P value**
Hemoglobin	.058	-.080	.330	.232
Sex of child	-.043	-.761	.288	.375
Gestational age	-.136	-.309	-.054	.005
Malaria infection status	.013	-.672	.892	.782
		***95*% *CI for predicting fetal cord malaria parasitemia***		**P value**
	***Odds ratio***	**Lower**	**Upper**	
Malaria Status	3.808	1.916	7.571	<0.001
Sex	1.008	.984	1.033	.497
Maternal anemia	1.035	.566	1.892	.910
Nine of more years of education	.863	.418	1.778	.689

## Discussion

This study presents data that are suggestive that newborns of women infected with HIV and/or malaria are at higher risk of fetal anemia and cord blood malaria parasitemia. Fetal cord hemoglobin levels were positively associated with gestational age and there is a link between maternal malaria parasitemia and fetal cord anemia in our study sample. HIV-malaria co-infection is perceptibly a strong predictor of adverse perinatal outcomes and associated morbidity in neonates in the study area. Prevalence of maternal and fetal anemia among HIV+ women were 78.6% and 40% respectively. The prevalence of anemia in pregnancy varies considerably [28,29]. In relations to the findings of McLean [30], in which the prevalence of anemia among pregnant African women was estimated to be 55%, our prevalence of 78% could be attributed to the additional effect of HIV infection. Several other studies have found high prevalence of fetal anemia and anemia in pregnancies particularly in sub-Saharan Africa and this have been attributed partly to diseases such as malaria and HIV/AIDS [3,29]. Adjusted odds ratio for the effect of malaria on fetal anemia was found to be 1.41 (95% CI, 1.05 - 1.90) in this current study. According to Griffin *et al*., [31] malaria parasites tend to have high preference for the placenta for their replication, evidenced by their proliferation and accumulation in the placenta, thereby infecting red blood cells of the placenta and reducing fetal hemoglobin levels. Abrams *et al*., [32] however found that malaria infection was associated with low anemia among pregnant mothers but not fetal hemoglobin. Uneke [27] also found that although prevalence of fetal anemia among women infected with malaria was higher compared to that of infants born to malaria uninfected women, the difference was not statistically significant. The complex and multifactorial influences on fetal anemia have been suggested as some of the reasons behind this discrepancy in literature [18].

High prevalence of maternal and infant anemia in this study can however be attributed largely to the synergistic effects of HIV and malaria co-infection. In as much as the study was conducted among HIV positive mothers thereby reducing the effect of HIV infection on study outcome, the high proportion of immuno-compromised mothers with CD4-counts < 350 cells/mm^3^ (Table 2) cannot be ignored. In pregnant women, co-infection with HIV and malaria have been associated with several negative outcomes in newborns including low birth weight (LBW) attributed largely to low fetal hemoglobin levels [33-35]. Several studies have also reported positive associations between HIV infection and higher levels of malaria parasitemia [13,36,37] where the immuno-compromising effect of HIV infection, leaves malaria parasites to infect red blood cells. This causes maternal anemia by decreasing maternal blood output, and also affecting exchange of materials between the mother and fetus [20-23]. Such changes may interfere with the maternal-fetal nutrient and metabolite exchanges, impairing iron transmission from mother to fetus thereby resulting in fetal anemia [21,23]. Uneke [27] observed that the prevalence of fetal anemia was considerably higher among babies born to HIV-positive mothers compared to those of HIV-negative mothers. Infant anemia was also found to be worse in HIV-uninfected infants when born to HIV-seropositive mothers compared with those born to HIV-seronegative mothers [38]. Data paucity on the effects of HIV infection on fetal anemia is a problem, particularly on the mechanisms although varieties of significant and positive associations have been found in some sub-Saharan African countries where HIV is endemic [27,38].

Fetal anemia was found to be significantly associated positively with maternal hemoglobin levels [0.29 (0.03 – 0.55)]. This indicates that the anemic status of mothers have some influence on that of their infants at delivery. These observations are consistent with studies that found high prevalence of fetal anemia in sub-Saharan African countries particularly in malaria-endemic regions [3,27,39]. Low maternal hemoglobin at delivery was also found to be a major factor associated with fetal anemia among southern Malawi women [3]. Recent studies outside the sub-Saharan African region have also found positive associations between maternal and infant hemoglobin and serum ferritin levels [35,40].

In a study among pregnant women in Bangladesh, Akhter *et al*., [35] found that [LOW] maternal hemoglobin due to iron deficiency (serum ferritin) correlated positively with cord ferritin (r=0.94; p<0.001) and had significant adverse effects on fetal outcome, including placental weight and birth weight. In contrast to the findings of this study, other studies conducted both within and outside Africa have found negative or no significant associations between maternal and infant anemia status [27,32,40,41]. Erdem *et al*., [41] observed from their study in Turkey that cord hemoglobin and mean corpuscular volumes were not affected by maternal anemia status. According to them, high serum erythropoietin levels were associated with low maternal hemoglobin levels and suggested that maternal anemia rather induces fetal erythropoiesis. Abrams *at al*., [32] also found from their study conducted in Malawi that cord hemoglobin levels did not correlate to maternal hemoglobin concentration. In a related study, Shao *et al*., [40] also noted that despite widespread maternal iron deficiency among pregnant Chinese women, iron nutrition seemed to meet fetal needs except when mothers were very iron deficient (serum ferritin levels below a threshold of 13.6 μg/L (β = 2.4; P = 0.001).

### Gestational age and cord blood hemoglobin concentration

Fetal cord hemoglobin was observed to be positively associated with gestational age with marginal significance [0.21 (−0.01, 0.43)]. It is logical to suppose that gestational age rather has an influence on fetal cord hemoglobin as development of red blood cells in the infant is progressive over the gestation period. This result is supported by the observation that fetal hematocrit increases with gestational age [42]. This corroborates results from other studies which indicated that critical changes that ensure development of red blood cells in the fetus occur in the last few weeks of pregnancy (third trimester) and reasonably explains why preterm babies may be more prone to infant anemia than full term babies [43]. In utero, fetal and maternal erythropoiesis occurs independently of each other. Although the placenta may play a role, the factors responsible for fetal red blood cell production are generally regarded to be produced by the fetus with erythropoietin; mainly produced by the liver, as the major growth factor responsible for fetal and neonatal erythropoiesis [44].

In the last few weeks of pregnancy (about 32 weeks) matured organs including the kidneys and bone marrow take over the process of erythropoiesis thereby predisposing premature infants whose organs may not be fully matured to fetal anemia [43].

### Sex of newborn versus fetal anemia and cord malaria

Fetal cord hemoglobin status was negatively associated with female gender of infant [−1.05 (−1.66, -0.44)] (Table 3 and Table 4). This suggests that female infants were at a greater risk of fetal anemia or anemia in infancy than boys. There is lack of studies addressing gender differences and their association with anemia and iron status right at birth even though some studies have been done in infants in the early months of life. According to Cassady [45] all infants experience a reduction in hemoglobin and erythropoietin production in the first 3 months after birth: the latter due to transition from a relatively hypoxic state in utero to a relatively hyperoxic state with increased tissue oxygenation. Considering that both hemoglobin and erythropoietin are affected, the findings of studies aimed at determining gender associations with anemia among full term infants particularly in the early months can be used to discuss the observations of this study since they may reflect the iron stores [46]. There is a growing body of evidence indicating that boys are at greater risk of anemia in infancy than girls and these are in contrast to the findings of this study [46].

In a recent study by Yang *et al*., [47] they found that even though iron deficiency anemia is uncommon among fully breastfed infants with a birth weight > 2500 g, male infants stood a higher risk of iron deficiency and iron deficiency anemia than female infants. Relatively high erythropoietic activity in boys during fetal life, smaller iron stores in boys despite their higher birth weight, and the possibility of boys experiencing higher intestinal losses than girls have been put forward as some of the likely explanations for the observed high risk of iron deficiency anemia in boys than girls during infancy [46]. Nevertheless, further studies are required in this field considering that fetal anemia and anemia of infancy are influenced by multiple factors, and in this study, HIV-infection and malaria, and genetics may play major and minor roles in complicating the association between gender and anemia at birth, and during the early years of infancy. Knowledge in this area may also be useful in formulating strategies and interventions aimed at protecting infants from anemia as early as at birth. Infant cord malaria on the other hand was observed to be positively associated with female gender [1.97 (1.06, 3.66)] (Table 3).

### Study strengths and limitations

This study is one of the few in Ghana that have attempted to evaluate the association between HIV on newborn health indicators, taking into account the effect of malaria. Of 1,154 pregnant women who were recruited into the study, about 30% were lost to follow-up (LTFU) before delivery. It is, however, worth noting that in terms of age distribution, gestation length at first antenatal visit, occupation, and socio-economic stratification, those women on whom follow up data were available did not differ significantly from those who were lost to follow-up. Also speculating that the baseline clinical outcomes of this group of women on whom data at delivery were available might differ from those who did not come back to deliver at the study hospitals a similar comparison was made. This analysis again showed that, with the exception of severe anemia at recruitment, the two groups of study participants were comparable (data not shown). On this premise, we proceed to discuss the study findings with confidence that the women on whom the main analysis were based, to a large extent represent the women enrolled into the study.

We, however, acknowledge though that, if an intention to treat analysis had been done, possibly different conclusions would have been made. Nevertheless, this approach, which works, on the assumption that none of those lost to follow-up suffered the adverse outcomes of interest can open the door to a misleading presentation of study results. It is also worthy of note that, the other alternative strategies available in dealing with this problem by imputing outcomes to those lost to follow-up, in general, all make unverifiable assumptions that may introduce bias in the estimates of treatment effect. We also acknowledge that several variables including timing of cord clamping could affect the fetal hemoglobin levels. Time of cord clamping was not documented in our study.

## Conclusions

Our data show that newborns of women infected with HIV, and/or malaria are at increased risk of fetal anemia and also cord blood parasitemia. Fetal cord hemoglobin levels were positively and significantly associated with gestational age and with maternal anemia. This may be uncovering an influence of maternal anemia and the anemic status of infants at delivery. The high level of maternal and infant anemia observed in this study could be attributable to the synergistic effect of HIV and malaria co-infection. Prevention of malaria infection during pregnancy may reduce the incidence of these adverse perinatal outcomes among HIV-positive women.

### Recommendations

In the settings where both malaria and HIV are co-endemic, routine screening of pregnant women for both malaria and HIV at first antenatal visits and intensification of the education of women at antenatal clinics on adverse perinatal outcomes attributable to malaria in pregnancy may be useful remedying actions. Also, deployment of the various means of preventing malaria, and anemia such as the use of insecticide treated nets and the use of intermittent preventive treatment (IPT) could equip women with self-protection tools. Finally, the promotion and encouragement of diets rich in iron and folic acid may reduce the incidence of anemia among this group of women and ultimately among newborns.

## Competing interests

The authors declare that they have no competing interest.

## Author’s contributions

AKL, WKA, IAQ, JT conceived, designed, implemented the research. AKL, FG, YA, IS, BN, JA, ASL contributed to the data analysis, and drafted different components of the manuscript. All the other authors contributed equally in manuscript proofreading and finalization. All authors read and approved the final manuscript.
